# Bringing CCM into a dish: cell culture models for cerebral cavernous malformations

**DOI:** 10.1515/medgen-2021-2091

**Published:** 2021-12-03

**Authors:** Dariush Skowronek, Robin A. Pilz, Konrad Schwefel, Christiane D. Much, Ute Felbor, Matthias Rath

**Affiliations:** Department of Human Genetics, University Medicine Greifswald, Greifswald, Germany; Interfaculty Institute of Genetics and Functional Genomics, University of Greifswald, Greifswald, Germany; Department of Human Genetics, University Medicine Greifswald, Fleischmannstraße 43, D-17475 Greifswald, Germany

**Keywords:** cerebral cavernous malformations, CRISPR/Cas9 genome editing, human endothelial cells, cell junctions, spheroid sprouting

## Abstract

Cerebral cavernous malformations (CCMs) are vascular lesions that can cause severe neurological complications due to intracranial hemorrhage. Although the CCM disease genes, *CCM1*, *CCM2*, and *CCM3*, have been known for more than 15 years now, our understanding of CCM pathogenesis is still incomplete. CCM research currently focuses on three main disease mechanisms: (1) clonal expansion of endothelial cells with biallelic inactivation of *CCM1*, *CCM2*, or *CCM3*, (2) recruitment of cells with preserved CCM protein expression into the growing lesion, and (3) disruption of endothelial cell–cell junctions in CCMs. We here describe novel CRISPR/Cas9-based *in vitro* models of CCM and discuss their strengths and limitations in the context of high-throughput drug screening and repurposing approaches.

## Introduction

**Figure 1:**
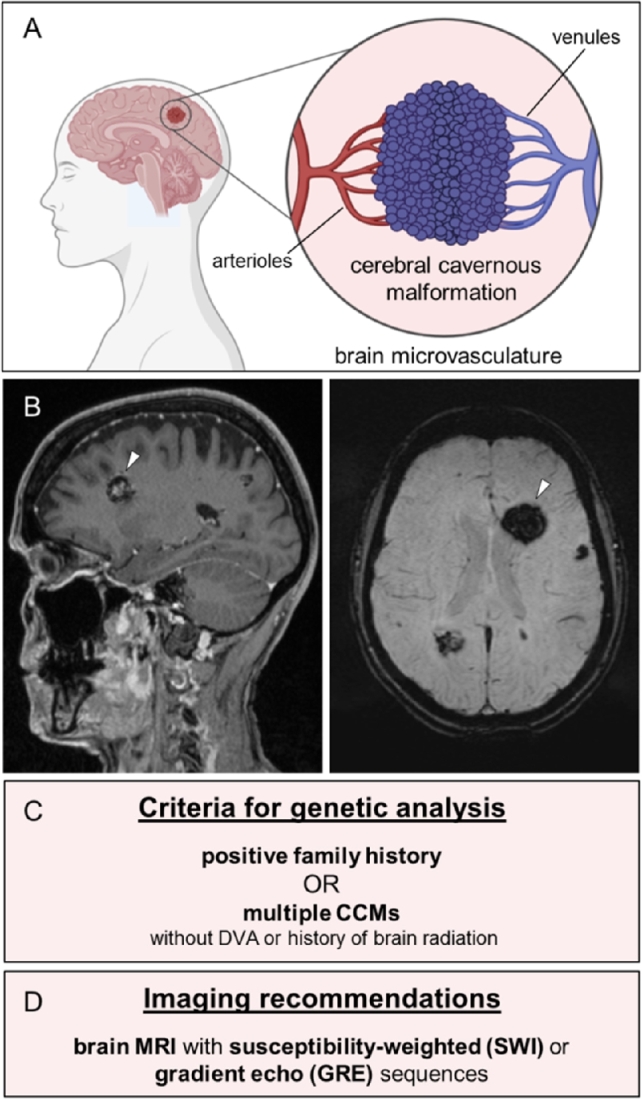
Clinical features of CCMs. (A) CCMs are mulberry-like vascular lesions in the microvascular bed of the central nervous system. (B) Sagittal T1-weighted (left) and axial susceptibility-weighted magnetic resonance images (right) show a large CCM (white arrowhead) and multiple smaller CCMs in both hemispheres of a patient with a pathogenic *CCM1* germline variant. (C) Criteria for genetic testing of patients with CCMs (adapted from [2]). DVA = developmental venous anomaly. (D) Recommended imaging techniques for diagnosis or follow-up of CCMs (adapted from [2]).

Cerebral cavernous malformations (CCMs) are mulberry-like lesions in the microvasculature of the central nervous system (Fig. 1A,B) which are found with a prevalence of 0.5 % in the general population. They consist of densely packed, thin-walled, and leaky endothelial channels. Depending on their location and size, they can lead to a diverse spectrum of clinical signs and symptoms. While many of these vascular malformations are asymptomatic, some cause focal neurological deficits, epileptic seizures, and stroke-like symptoms due to intracranial hemorrhage. Especially brainstem lesions may lead to significant neurological complications [1]. However, CCMs can not only manifest with symptomatic hemorrhage but also with nonhemorrhagic focal neurological deficits.

Besides sporadic cases, about 6–7 % of CCMs occur in a familial form that is inherited in an autosomal dominant manner and caused by loss-of-function germline variants in *CCM1* (*KRIT1*; OMIM: *604214), *CCM2* (OMIM: *607929), or *CCM3* (*PDCD10*; OMIM: *609118) [3], [4]. Familial cases usually become symptomatic in the fourth to fifth decade of life [5]. In this context, CCMs can be a significant cause of neurologic morbidity in middle-aged adults. Genetic counseling and testing should be offered to patients with a positive family history or multiple CCMs (Fig. 1C). For patients without a positive family history, however, current best practice guidelines only recommend genetic testing if there is no associated developmental venous anomaly (DVA) and no history of brain radiation, as these features usually indicate a sporadic case [2]. Magnetic resonance imaging (MRI) with susceptibility-weighted (SWI) or gradient echo (GRE) sequences is essential for making the correct diagnosis [2], [6] (Fig. 1D). Especially small CCMs can often only be detected with these special imaging techniques. Predictive genetic testing in children is possible because the results may guide the decision to perform an MRI examination, which may require sedation in young children [2].

Although more than 20 years have passed since the first disease gene, known as *CCM1* or *KRIT1* [7], [8], was identified, there is still no specific or targeted therapy for CCM patients. While symptomatic and easily accessible lesions may be treated with neurosurgical resection, conservative management is often the only option for patients with cavernous malformations in eloquent areas. Therefore, finding new pharmaceutical targets is a primary goal of CCM research. CCM studies in mice have recently added the mTOR inhibitor rapamycin and the third-generation tyrosine kinase inhibitor ponatinib to the short list of potential novel therapies [9], [10]. Unfortunately, *in vivo* studies are time consuming, expensive, and complex. Simplified *in vitro* systems are not perfect disease models either, but they can be used to study specific aspects of CCM pathobiology in more detail. Since they are less complex, less expensive, and compatible with the 3R principle (namely replacement, reduction, and refinement of animal experiments), they also qualify as first-line approach in high-throughput drug discovery studies. In the second or third line, *in vivo* models can then be used to validate novel drug candidates. It is important to realize that patients with sporadic CCMs could also benefit from new pharmacological treatments identified in those combined *in vitro*/*in vivo* screening assays because a substantial number of sporadic cases is caused by biallelic somatic *CCM1*, *CCM2*, or *CCM3* mutations [11], [12].

This article reviews the currently available CCM cell culture models and illustrates their strengths and limitations. In particular, we focus on our recent efforts to establish new CRISPR/Cas9-based *in vitro* models of CCM disease.

## Modeling the clonal expansion of mutant endothelial cells in CCMs

In efforts to find a treatment that can block disease progression, hope rests on a better understanding of the molecular mechanisms that trigger CCM formation. In reminiscence of Knudson’s two-hit model for retinoblastoma [13], DNA sequencing and immunohistochemical analyses of human CCMs demonstrated that *CCM1*, *CCM2*, or *CCM3* gene expression is completely inactivated by a germline and a second somatic mutation or by two somatic mutations in many cavernous malformations [10], [12], [14], [15], [16], [17]. However, the vascular lesions do not only consist of mutant endothelial cells. Instead, a mosaic pattern of mutant endothelial cells and heterozygous or wild-type cells is found in CCM mouse models and human CCM tissue samples of familial and sporadic cases, respectively [17], [18], [19]. How these cells interact and whether the mosaic state is necessary for the survival of mutant cells *in vivo* is not yet understood.

**Figure 2:**
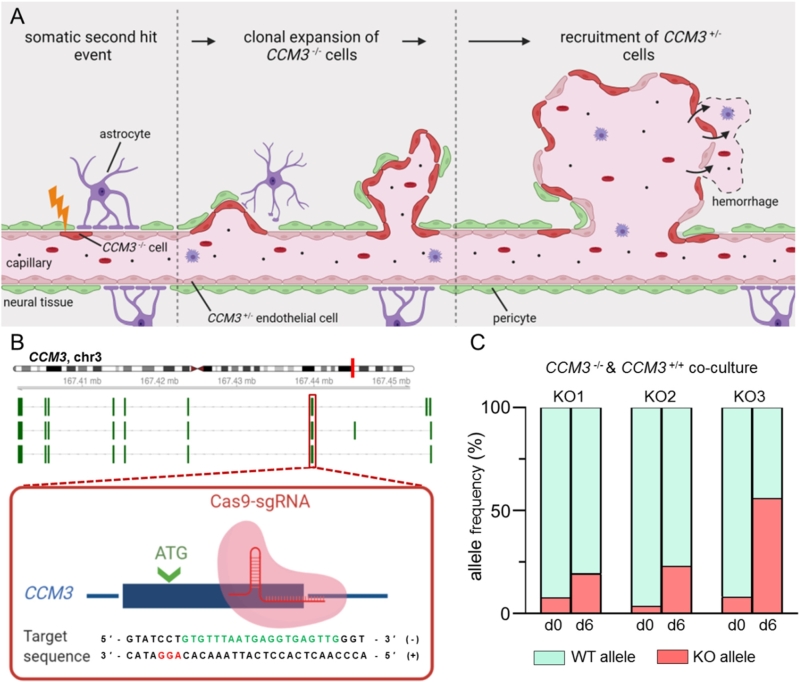
CCM3 gene disruption promotes clonal expansion of endothelial cells. (A) In patients with a *CCM3* germline mutation (${\mathrm{CCM}3}^{+/-}$), a second somatic *CCM3* mutation in an endothelial cell (${\mathrm{CCM}3}^{-/-}$) initiates CCM formation. A ${\mathrm{CCM}3}^{-/-}$ mutant endothelial cell undergoes clonal expansion and forms a CCM that is characterized by endothelial mosaicism of ${\mathrm{CCM}3}^{-/-}$ and ${\mathrm{CCM}3}^{+/-}$ endothelial cells. The impaired endothelial barrier function can lead to bleeding into the surrounding brain tissue. (B) *CCM3* knockout endothelial cells were generated with CRISPR/Cas9 genome editing. Biallelic loss-of-function variants were introduced into the first coding exon of *CCM3* (knockout [KO] clones 1 and 2: c.[87_88insAG];[87_88insAG] [p.[Phe30Serfs*5];[Phe30Serfs*5]]; KO3: c.[90dupT];[87_88insAGTTGGATAAACATGTTTATCCAACT] [p.[Asn31*];[Phe30Serfs*13]]). (C) ${\mathrm{CCM}3}^{-/-}$ CI-huVECs demonstrated significant expansion in co-culture with ${\mathrm{CCM}3}^{+/+}$ CI-huVECs. Knockout and wild-type (WT) allele frequencies were determined by amplicon deep sequencing after six days of co-culture.

Using CRISPR/Cas9 genome editing, we were recently able to study the effects of the second hit in blood outgrowth endothelial cells (BOECs) of a CCM patient with a pathogenic *CCM1* germline mutation. Signs of endothelial dysfunction, namely the disruption of intercellular junctions, the formation of actin stress fibers, and the upregulation of the transcription factor KLF2, were only observed after inactivation of the second *CCM1* allele [20], [21]. Interestingly, we were able to model a phenomenon *in vitro* that has recently been observed in CCM mouse models: clonal expansion of mutant endothelial cells [18], [19]. ${\mathrm{CCM}1}^{-/-}$ BOECs and ${\mathrm{CCM}3}^{-/-}$ immortalized human umbilical vein endothelial cells (CI-huVECs) demonstrated a striking survival advantage when co-cultured with ${\mathrm{CCM}1}^{+/-}$ BOECs or ${\mathrm{CCM}3}^{+/+}$ CI-huVECs (Fig. 2A–C), respectively [21], [22]. We also noticed resistance of ${\mathrm{CCM}1}^{-/-}$ BOECs and ${\mathrm{CCM}3}^{-/-}$ CI-huVECs to apoptosis, a feature reminiscent of malignant tumors. Even treatment with the broad-spectrum protein kinase inhibitor staurosporine, a potent inducer of apoptosis, only led to minimal activation of caspase-3 in CCM1- and CCM3-deficient cells [21], [22]. In a proof-of-principle approach, CRISPR/Cas9 genome editing also enabled us to study the feasibility of a targeted gene repair. While we were able to correct the *CCM1* germline mutation in a significant number of ${\mathrm{CCM}1}^{+/-}$ BOECs *in vitro*, corrected ${\mathrm{CCM}1}^{+/+}$ BOECs were replaced by highly proliferative ${\mathrm{CCM}1}^{-/-}$ BOECs in co-culture [21]. In human CCM, where CRISPR/Cas9-mediated gene repair would not eradicate all mutant endothelial cells, the therapeutic benefit of such a genome editing approach would therefore be limited.

The new hypothesis that the tumor-like behavior of mutant endothelial cells represents a suitable therapeutic target has also been supported by the detection of *PIK3CA* mutations in CCMs [10]. The identification of somatic variants in this well-known oncogene suggests a three-hit mechanism in CCM pathogenesis. Following this intriguing model, only the combination of inactivating mutations in *CCM* genes acting as vascular “suppressor genes” and activating variants in vascular “oncogenes” can provoke a severe or aggressive course of CCM disease [10].

## Cell culture models of the endothelial barrier dysfunction in CCMs

**Figure 3:**
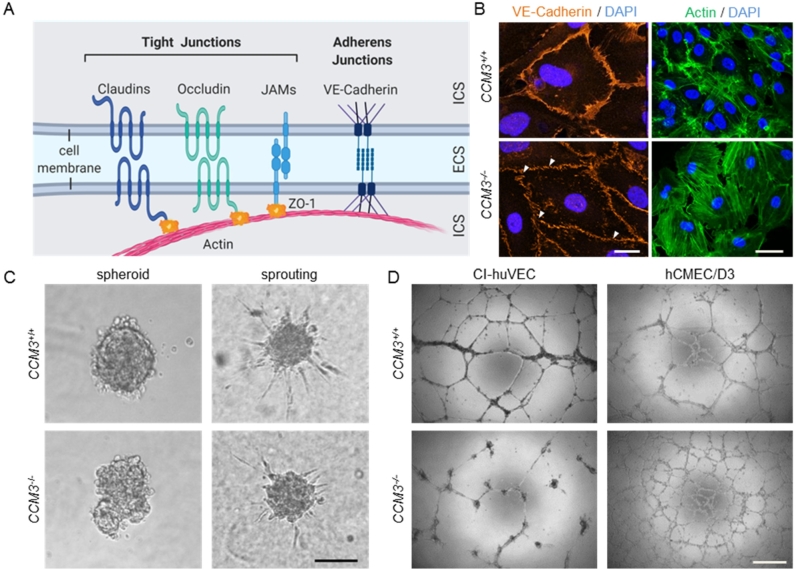
Disorganized cell junctions and impaired function in 3D models of angiogenesis upon *CCM3* gene inactivation. (A) Scheme of endothelial tight and adherens junctions. ICS = intracellular space. ECS = extracellular space. ZO-1 = zonula occludens protein 1. JAM = junctional adhesion molecule. (B) In contrast to wild-type CI-huVECs, ${\mathrm{CCM}3}^{-/-}$ CI-huVECs demonstrated numerous small gaps (white arrowheads) and a less homogeneous pattern in VE-cadherin staining (red). Scale bar $\overset{\wedge }{=}$ 20 µm. They also displayed significant actin stress fiber formation (green, phalloidin staining). Scale bar $\overset{\wedge }{=}$ 25 µm. DAPI (blue) was used to stain cell nuclei. (C) ${\mathrm{CCM}3}^{-/-}$ CI-huVECs demonstrated impaired spheroid formation and VEGF-induced sprouting. The number and length of sprouts formed by ${\mathrm{CCM}3}^{-/-}$ spheroids upon stimulation with 25 ng/ml VEGF-A were significantly reduced. Scale bar $\overset{\wedge }{=}$ 100 µm. (D) *CCM3* gene inactivation had cell type-specific effects on endothelial tube formation on Matrigel. While tubes formed by ${\mathrm{CCM}3}^{-/-}$ CI-huVECs were unstable and had fallen apart 17 h after seeding on Matrigel, ${\mathrm{CCM}3}^{-/-}$ hCMEC/D3 cells formed more stable meshes than hCMEC/D3 wild-type controls. Scale bar $\overset{\wedge }{=}$ 500 µm.

Apart from blocking CCM formation, restoration of an intact endothelial barrier is another primary objective in CCM therapy. As part of the blood–brain barrier (BBB), vascular endothelial cells participate in the tightly regulated exchange of ions, molecules, and cells between the blood and the brain [23]. However, no targeted therapies have yet been approved to prevent CCM bleeding and hemorrhage-associated neurological complications.

Endothelial tight and adherence junctions are indispensable to maintain BBB integrity but are highly dysfunctional in CCMs. Claudins, occludin, and junctional adhesion molecules (JAMs) are major components of endothelial tight junctions. These transmembrane proteins are linked to the actin cytoskeleton by scaffold proteins like zonula occludens protein 1 (ZO-1) (Fig. 3A). Destabilization of tight junctions and reduced expression of claudin-5, occludin, and ZO-1 have been observed in human CCM tissues [24], [25] and CCM mouse models [26], [27], [28]. These features of CCM disease can be perfectly reproduced *in vitro* [25], [28]. However, not only tight junctions but also adherens junctions are disorganized in CCMs. Using CRISPR/Cas9 genome editing, we could mimic disruption of adherens junctions in ${\mathrm{CCM}3}^{-/-}$ CI-huVECs (Fig. 3B) and ${\mathrm{CCM}1}^{-/-}$ BOECs [20]. In line with previous literature reports [21], [22], [29], [30], [31], the dysfunction of endothelial cell–cell junctions was accompanied by an increased formation of actin stress fibers (Fig. 3B). These phenotypes are useful surrogate markers for the hyperpermeability and increased bleeding risk of CCMs.

## Three-dimensional cell culture models in CCM research

The impaired interaction of mutant endothelial cells can also be visualized in three-dimensional (3D) cell culture models. More than 20 years ago, Thomas Korff and Hellmut G. Augustin developed a 3D spheroid formation and sprouting assay to analyze endothelial cell differentiation, cell–cell and cell–matrix interactions, and capillary sprouting [32], [33]. Using this assay, we demonstrated that CI-huVECs could only form irregular and barely demarcated spheroids upon CRISPR/Cas9-induced *CCM1*, *CCM2*, or *CCM3* gene disruption [22], [29]. Furthermore, *CCM3* gene inactivation in CI-huVECs and the immortalized human brain microvascular endothelial cell line D3 (hCMEC/D3) significantly impaired sprouting (Fig. 3C) [22]. Since transient *CCM3* knockdown and genetic *CCM3* knockout modulate this fundamental process differently [22], [26], compensatory mechanisms likely influence the angiogenic behavior of ${\mathrm{CCM}3}^{-/-}$ endothelial cells.

Disruption of endothelial junctions after *CCM* inactivation has also been found in transwell permeability assays. Upon CCM1, CCM2, or CCM3 depletion, the permeability of HUVEC monolayers was significantly increased [31], [34]. These results demonstrate that not only an altered 3D organization and angiogenic behavior of mutant cells, but also the leaky phenotype seen in CCMs can be modeled in *in vitro* systems. However, it can be sometimes challenging to directly compare the results of different *in vitro* models. An example is endothelial tube formation of mutant endothelial cells on Matrigel, which is another widely used *in vitro* angiogenesis assay. ${\mathrm{CCM}3}^{-/-}$ CI-huVECs form endothelial tubes that rapidly disintegrate (Fig. 3D) [22], a phenomenon that has also been reported for primary HUVECs after short hairpin RNA-mediated knockdown of *CCM1* and *CCM2* [35]. In contrast, ${\mathrm{CCM}3}^{-/-}$ hCMEC/D3 cells were able to form stable tubes on Matrigel (Fig. 3D). The different behavior on Matrigel might be a cell type-specific effect related to the fact that CI-huVECs and hCMEC/D3 cells are derived from endothelial cells from different vascular beds. However, an effect of different culture media and supplement concentrations cannot be excluded either. A combination of different assays is therefore the best way to obtain valid results.

## High-content screening in CCM drug discovery

Since drug discovery and development studies are time consuming and cost-intensive, drug repurposing approaches have become popular in recent years. In the context of CCM, endothelial barrier function and cell proliferation assays have already been used successfully in drug repurposing screens. Gibson and colleagues defined the reversion of VE-cadherin disassembly and actin stress fiber formation in *CCM2*-silenced human dermal microvascular endothelial cells as primary read-out parameters [36]. Using *in vitro* transcellular resistance analyses, dermal permeability assays in inducible endothelial-specific *Ccm2* knockout mice, and magnetic resonance imaging as secondary, tertiary, and quaternary screens, they identified tempol and cholecalciferol as promising candidates for CCM therapy [36]. Nishimura and colleagues also used a multi-step screening approach [37]. Drugs that could inhibit the proliferation of CCM3-deficient mouse astrocytes were validated in an RNAi-based *Drosophila* model and two mouse models of CCM disease. With this screening strategy, the authors identified the combination of fluvastatin and zoledronate to be effective *in vivo* and *in vitro* [37]. Finally, Otten and colleagues used *ccm2* mutant zebrafish embryos, *kri-1* (*CCM1*), and *ccm-3* ablated *C. elegans*, as well as *CCM2* knockdown HUVECs in a multi-organism-based screening approach. In downstream analyses, they validated that indirubin-3-monoxime treatment rescued VE-cadherin and actin phenotypes in *CCM1-*, *CCM2-*, and *CCM3-*silenced HUVECs [38].

Another positive example of drug repurposing in the context of CCM disease is propranolol. This pleiotropic β-blocker has recently been shown to reduce lesion burden in CCM mouse and zebrafish models [39]. A further study demonstrated that propranolol treatment also increased pericyte coverage and prevented vascular leakage in inducible endothelial-specific *Ccm3* knockout (CCM3^iECKO^) mice [40]. After encouraging case reports on propranolol treatment in CCM patients, its effectiveness is now assessed in the Treat_CCM study, a multicenter, open-label, randomized trial [41].

Drug repurposing and discovery studies have relied on *Ccm* knockout mouse models or RNAi-based *in vitro* gene knockdown models in human endothelial cells so far. Both have strengths and limitations. In particular, discrepancies between transient gene knockdowns and genetic knockouts, as well as the limited predictive value of some mouse studies for humans are inherent weaknesses of these studies. Because they are easy-to-handle, cost-effective, and 3R-compliant, the use of novel human CRISPR/Cas9-based *in vitro* models in primary screens may help to accelerate the process of finding effective drugs for CCM patients.

## Co-culture and iPSC-based CCM models

Notably, several studies have disclosed that CCM formation and disease progression are caused by more than just endothelial dysfunction. Pericytes which interact with the abluminal side of endothelial cells and astrocytic endfeet which enclose blood vessels are also major components of the BBB and participate in CCM pathogenesis [42], [43], [44]. Wang and colleagues, for example, demonstrated that specific *Ccm3* deletion in mural cells induces a CCM phenotype in mice [44]. In particular, they found reduced cell spreading and migration of CCM3-deficient pericytes which caused impaired association with endothelial cells [44]. Additionally, a recent publication highlighted the crosstalk between endothelial cells and astrocytes in CCM lesion development. Increased endothelial NO synthase (eNOS)/nitric oxide (NO)-dependent signaling in dysfunctional endothelial cells leads to elevated levels of the astrocyte-derived angiogenesis factor VEGF, which contributes to endothelial cell junction disassembly linked to an increased risk of hemorrhage [43]. These observations provide a first explanation of why CCMs only arise in the central nervous system, although the CCM proteins are ubiquitously expressed. They also suggest that endothelial monocultures may not adequately illustrate CCM disease. *In vitro* co-culture models of endothelial cells, astrocytes, and pericytes may be more suitable. Patient-specific induced pluripotent stem cells (iPSCs) and their direct differentiation into all three cell types may allow the development of new co-culture models in the future. Co-culturing these cells in transwell or microfluidic models can improve the barrier properties and allows studying BBB dysfunction in an isogenic system [45]. Measuring the transendothelial electrical resistance (TEER) of knockout BBB co-cultures in a compound library screen may help to find a drug that can reduce the bleeding risk of CCMs. A fascinating direction of CCM disease modeling might also be the combination with a CCM xenograft model [46]. Implantation of human spheroid co-cultures into murine models might reconstruct the *in vivo* cellular environment of CCM by keeping the human origin of the affected cells.

## Outlook

Although there will be no “one-stop shopping” in CCM drug discovery in the near future, we now have a broad toolkit of *in vitro* models to study CCM pathogenesis and search for new CCM therapies. In particular, CRISPR/Cas9 genome editing has become an invaluable tool to model fundamental cellular and molecular processes of CCM formation, disease progression, and endothelial barrier disruption. Targeted gene inactivations in endothelial cells, pericytes, and astrocytes will facilitate more complex 3D co-culture models of CCM. Combined with live-cell imaging and new CRISPR tools, e. g., CRISPR activation (CRISPRa) or CRISPR interference (CRISPRi), these models will help to understand the dynamics of BBB dysfunction in CCMs better. However, higher complexity is usually accompanied by lower compatibility with high-throughput drug screening assays. Therefore, future efforts to model CCM disease *in vitro* will likely go into two directions: (1) simple but high-throughput-compatible *in vitro* assays and (2) complex cell culture models to closely mimic the *in vivo* situation.
